# Roles of lactylation in lipid metabolism and related diseases

**DOI:** 10.1038/s41420-025-02705-4

**Published:** 2025-08-23

**Authors:** Bing Zhao, Zhuoqing Lan, Caixia Li, Hao Wang

**Affiliations:** 1 Department of Anesthesiology, the First Affiliated Hospital, Zhejiang University School of Medicine, Hangzhou, China; 2 Department of General Practice Medicine, the Fourth Affiliated Hospital, Zhejiang University School of Medicine, Yiwu, China; 3 Department of Neurology, Tongde Hospital of Zhejiang Province, Hangzhou, China

**Keywords:** Lipids, Post-translational modifications, Metabolic disorders

## Abstract

Lipids are indispensable molecules that play key roles in cell physiology by acting as structural elements, energy reservoirs, and participants in signal transduction. Recent studies have identified lactylation as a novel post-translational modification crucial for maintaining cellular and tissue equilibrium. Research has shown that lactylation regulates the turnover of proteins and lipids integral to lipid metabolism. However, comprehensive reviews addressing the complex relationship between lactylation and lipid metabolism are lacking. In this review, we summarize current findings regarding the influence of lactylation on lipid metabolism and the regulatory mechanisms. Further exploration of the intricate mechanisms underlying the role of lactylation in lipid metabolism, alongside the development of lactylation-targeted therapies, could provide innovative approaches to manage diseases linked to dysregulated lipid metabolism.

## Facts

- Lactylation functions as a multifaceted regulator in major lipid metabolism pathways.
- Dysregulation of lactylation contributes to lipid abnormalities in various diseases.
- Targeting lactylation might provide precise therapeutic strategies for lipid metabolism-related diseases.

## Open questions

- What are the specific regulatory mechanisms for lactylation in different metabolic diseases?
- What regulatory crosstalk exists between lactylation and other PTMs in lipid metabolism reprogramming?
- The identified enzymes exhibit restricted specificity toward lactylation, giving rise to the challenges of developing more precise enzymatic tools to address this limitation.

## Introduction

Lactylation, initially discovered on nucleosomal histones, has emerged as a crucial mechanism in macrophages for maintaining immune homeostasis during bacterial attack [1]. This epigenetic modulation involves the covalent attachment of a lactyl-group to lysine residues in core histones, which neutralizes the positive charge of lysine residues and potentially alters histone–DNA interactions and chromatin accessibility, ultimately driving the transcription of downstream target genes [1, 2]. Lactylation preferentially targets nucleophilic sites like amino groups, enabling precise interactions with complementary functional groups, thereby inducing significant alterations in the physicochemical properties and biological functions of the modified molecules [3, 4]. Notably, the scope of lactylation extends beyond histones, and further research has revealed lactylation of non-histone proteins including signal transduction proteins and enzymes [3, 5, 6]. Lactylation on non-histone proteins has a wide variety of effects, spanning from modulating protein stability and enzyme activity to influencing their distribution, structure, and interactions [6, 7].

**Fig. 1:**
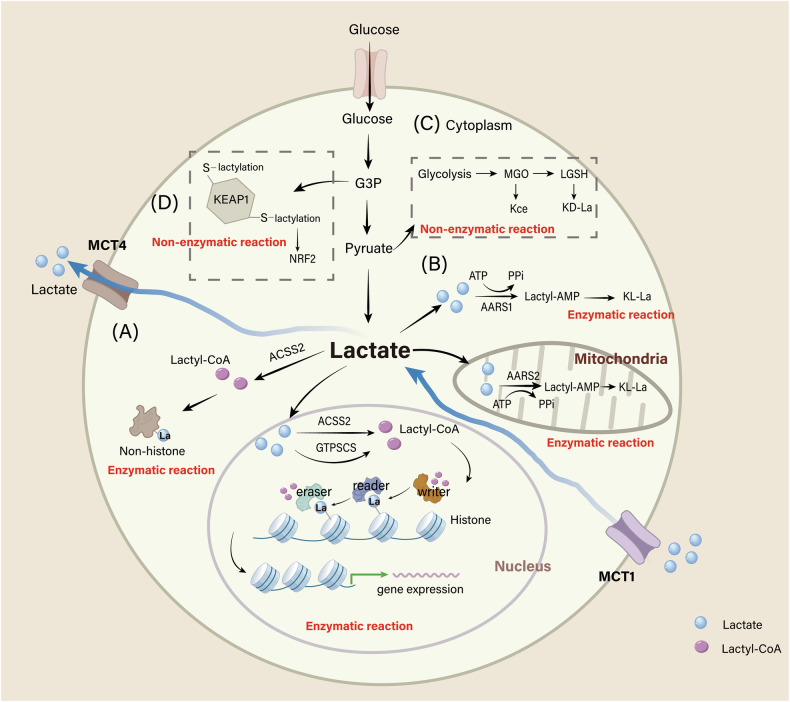
Different forms of lactylation. **A** L-lactate can be enzymatically converted into lactyl-CoA, which serves as a substrate for KL-la on both histones and non-histone proteins. **B** Alanyl-tRNA synthetases 1/2 (AARS1/2) function as lactyltransferases, catalyzing the formation of lactyl-AMP complex from L-lactate and ATP. Lactyl-AMP is covalently attached to lysine on target proteins. Notably, AARS1 is localized in the cytoplasm, whereas AARS2 resides in mitochondria. **C** The glyoxalase pathway intermediate LGSH represents a source of KD-la, driving a non-enzymatic transfer of the D-lactyl-group on lysine residues. Additionally, Kce is formed by a non-catalytic reaction between MGO and lysine residues of proteins. **D** Accumulation of glyceraldheyde 3-phosphate (G3P) induces non-enzymatic S-lactylation of cysteine residues in Kelch-like ECH-associated protein 1 (KEAP1), leading to activation of nuclear factor erythroid 2-related factor 2 (NRF2).

Recent studies identified lysine L-lactylation (KL-la), D-lactyl-lysine (KD-la), and N-ε-(carboxyethyl)-lysine (Kce) [8]. KL-la is characterized by a series of reversible enzyme-catalyzed reactions and is dynamically regulated by glycolysis and Warburg effects, with impacts on both histone and non-histone lactylation. This enzyme-dependent lactylation is dynamically regulated by writers, erasers, and readers, ensuring a balance between lactylation and delactylation [9–11]. Writers are enzymes or proteins that catalyze the addition of lactyl-groups, whereas erasers are those capable of removing lactyl-groups via hydrolysis. Readers specifically recognize and bind to lactyl-groups. These categories are outlined in Table 1. In contrast, KD-la stems from a non-enzymatic reaction between S-D-lactyl-glutathione (LGSH) and lysine residues, while Kce occurs between methylglyoxal (MGO) and lysine residues [12–14]. Moreover, a specific type of non-enzymatic, metabolite-derived cysteine lactylation (S-lactylation) has been identified as widely present in metabolic enzymes [15, 16] (Fig. 1).

**Table 1:** Writers, readers, and erasers of lactylation.

Writers	Erasers	Readers
1) KATs	1) Zn^2+^ dependent:	BRG1
P300/CBP family (P300, CBP)	Class I (HDAC1–3 and 8)	DPF2
GNAT family (GCN5/KAT2A, YiaC)	Class II (II b: HDAC 6)	TRIM33
MYST family (MOF/KAT8, TIP60/KAT5, HBO1/KAT7)	2) NAD^+^ dependent:	
2) AARSs family (AARS1, AARS2)	Class III (SIRT1–3, 6, and CobB)	
3) Others (ATAT1, ACAT2, ESCO2, NAA10, HDAC6)		

*KATs* Lysine acyltransferases, *CBP*CREB-binding protein, *GNAT* GCN5-related N-acetyltransferase, *MYST* Moz-Ybf2/Sas3-Sas2-Tip60, *TIP60* Tat-interactive protein 60, *KAT* (lysine acetyltransferase), *MOF* (males absent on the first), *HBO1* (histone acetyltransferase binding to ORC1), *ATAT1* (α-tubulin acetyltransferase 1), *ESCO2* (establishment of sister chromatid cohesion N-acetyltransferase 2), *NAA10* N-α-acetyltransferase 10, *AARS* alanyl-tRNA synthetase, *HDAC*histone deacetylase, *SIRTs* (Sirtuins), *TRIM33* (tripartite motif containing 33), *DPF2* (double PHD finger 2), *BRG1* (Brahma-related gene 1).

Collectively, lactylation plays an active role in cellular and biological processes, including gene regulation, metabolic orchestration, and immune responses [17, 18]. Aberrant lactylation is associated with multiple diseases, ranging from developmental anomalies [19, 20], cardiovascular disorders [21, 22], cerebral diseases [23, 24] to inflammation [25–28] and cancers [29–31]. Lactylation has attracted escalating attention, particularly in relation to lipid metabolism [17, 18]. It regulates lipid homeostasis under physiological conditions and also modulates disease progression through lipid metabolic pathways. Elucidating its specific roles and regulatory mechanisms could help identify lactylation markers to facilitate early diagnosis and disease monitoring. Furthermore, such research could provide potential therapeutic targets and drug candidates for addressing these disorders. In this comprehensive review, we systematically analyze the current findings on the roles of lactylation in mechanisms of lipid metabolism, aiming to provide a theoretical framework for future research and clinical translation.

## Lactylation-mediated control of fatty acid (Fa) metabolism

**Fig. 2:**
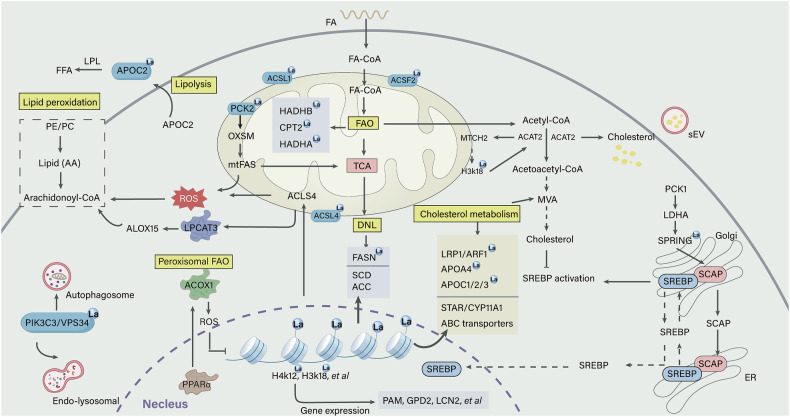
Summary of the mechanisms by which lactylation influences lipid metabolism. An overview of lactylation across major lipid metabolism pathways at multiple levels reveals that lactylation functions as a bidirectional regulator by dynamically regulating transcription factors, metabolic enzymes, and signaling pathways. In FA metabolism, FASN lactylation inhibits DNL, whereas H3K18la promotes FA synthesis by regulating enzymes including SCD and ACC. Furthermore, PCK2 lactylation enhances the production of mtFAS products. The differential HADHA lactylation not only promotes FAO, stimulates mitochondrial biogenesis and augments OXPHOS, but also inhibits its enzymatic activity, thereby impairing mitochondrial function and perturbing energy metabolism. CPT2 lactylation suppresses FAO and constrains OXPHOS, potentially leading to FFA accumulation, which subsequently activates PPARγ. PPARα initiates ACOX1 transcription, which in turn inhibits H3K18la via ROS-dependent PKM2 downregulation. APOC2 lactylation accelerates lipolysis, while Vps34 lactylation induces autophagy. In cholesterol metabolism, SPRING lactylation mediated by pPCK1-pLDHA axis enhances MVA pathway by promoting the retrograde transport of SCAP. The positive feedback loop involving lactate, H3K18la, and ACAT2 enhances cholesterol biosynthesis. ACAT2 stabilizes its own expression by MTCH2 acetylation, thereby reducing mitochondrial OXPHOS. Moreover, H3K18la-driven ACAT2 expression facilitates cholesterol secretion via sEVs, further contributing to immunosuppressive TME. Additionally, H3K18la promote progesterone synthesis. Lactylation of apolipoproteins facilitates the degradation of cholesterol-related proteins. Key lipid metabolism proteins that have been identified as targets for lactylation are indicated by the blue lactylation (La) circles.

FA homeostasis is a critical determinant of normal metabolic functions and is governed by FA uptake, de novo lipogenesis (DNL), FA oxidation (FAO), and other interconnected metabolic pathways. Recent evidence suggests lactylation is closely associated to numerous aspects of FA homeostasis [32–37]. As discussed in the following section, increasing evidence supports lactylation acting as a crucial regulator for each of these processes (Fig. 2).

### FA synthesis

FA synthase (FASN) is a rate-limiting enzyme in DNL pathway, and its activity and protein stability are modulated through lactylation [34]. FASN is identified as a differentially lactylated protein, containing three specific lactylation sites (Table 2). Malonyl/acetyltransferase (MAT) is a crucial functional region of FASN that mediates the transfer of acetyl-CoA and malonyl-CoA to acyl carrier protein (ACP) to initiate FA synthesis [38]. Lactylation of K673 in FASN, which is located in close spatial proximity to the catalytically active site of MAT domain, significantly inhibits FASN enzymatic activity, thereby alleviating DNL in hepatocytes [39]. Moreover, FASN lactylation is also closely associated with lipid synthesis stimulated by exercise and hyperlactylates at least at four lysine residues [33]. The elevated FASN lactylation in adipose tissue inhibits FASN activity and diminishes palmitate and triglyceride (TG) synthesis, suggesting the existence of a lactylation-dependent feedback loop during DNL [33].

**Table 2:** List of lactylation events that regulate lipid metabolism.

Substrates	Lactylation sites	Writer/Eraser	Functions	Health/disease	Refs
FASN	K528/673/1071	/	Modulates the MAT domain by lactylation, thereby suppressing FASN enzymatic activity and consequently reducing DNL	NAFLD, HCC, HIIT	[33–35]
APOC2	K70	P300/HADC3	Induces immunotherapy resistance by promoting extracellular lipolysis	NSCLC	[32]
CPT2	K457/458	AARS2/SIRT3	Integrates intracellular hypoxia and lactate to regulate OXPHOS, and induces follicle development	Exercise, POI	[37, 57]
SPRING	K82	KAT7	pAKT/pPCK1/pLDHA/SPRINGla axis induces ferroptosis resistance in AKT-hyperactivated ICC via MVA flux reprogramming.	ICC	[83]
HADHA	K519, K415, K644, K60, and K569	/	KDM6B-mediated HADHA demethylation/lactylation promotes FAO and mineralization	Periodontitis	[62]
HADHA	K166, K728	SIRT1/3	Disturbs cardiomyocyte mitochondrial function and energy metabolism	Septic myocardial depression	[65]
PCK2	K100	KAT8	Aggravates hepatic ferroptosis during IRI by OXSM-dependent mtFAS reprogramming	LT	[44]
Histone H3	K18	/	Promotes HCC via lipid metabolism remodeling	HCC	[45]
Histone H3	K18	/	PPARα activation reverses acquired resistance to TMZ in GBM by inhibiting H3K18la	GBM	[61]
Histone H3	K12	/	H4k12la mediated by Fam172a in POMC neurons regulates lipid and glucose metabolism	Obesity	[49]
Histone H3	K18	P300	H3K18la-induced ACAT2/sEV-cholesterol axis orchestrates cholesterol-mediated immunosuppressive reprogramming	PC	[84]
Histone H3	K18	/	Promotes NAFLD via targeting METTL3/YTHDF1/SCD1 m^6^A axis	NAFLD	[48]
Histone H3	K18	/	Activates HIF1A and exacerbates SAP via ACSL4/LPCAT3/ALOX15 pathway-induced ferroptosis	SAP	[74]
Histone H3	K18	P300/CBP	H3K18la-driven mechanism induces ferroptosis via METTL3-mediated m^6^A modification	ALI	[72]
Histone H3	K18	P300/CBP/SIRT3	Induces ACSL4 transcription to activate ferroptosis	IVDD	[73]
GCLM	K34	ACAT2	Promotes ferroptosis resistance in KRAS^G12D^-mutant cancers	KRAS^G12D^ mutant cancers	[86]
Histone H3	K18	/	Induces hCG-induced luteinization in hypoxic GCs by upregulating CYP11A1 and STAR transcription	Luteal insufficiency	[85]
Histone H3	K18	/	Induces chondrocyte ferroptosis	OA	[135]
ACSL4	K412	P300/CBP/SIRT3	Activates ferroptosis	IVDD	[73]
ARF1	K73	/	Promotes LRP1-induced mitochondria release in astrocyte–neuron crosstalk	Stroke	[143]
NSUN2	K508	NAA10	Drives cancer resistance to ferroptosis by enhancing GCLC/GSH signaling	GC	[75]
Vps34	K356/K781	KAT5/TIP60	Increases lipid kinase activity and promotes autophagic flux and endolysosomal trafficking	Exercise, Cancer	[76]
RUBCN	K33	/	Promotes PI3P levels and LAP formation via interaction with Vps34	Infectious diseases	[78]
APOA4, APOC1, APOC3	/	/	Promotes downregulation of cholesterol-related proteins	Tendinopathy	[93]
Histone H4	K12	/	Promotes chemotherapy resistance by regulating ABCC2, ABCC3, and ABCC10	DLCC	[95]
Histone H3	K18	/	H3k18la-driven GPD2 regulates M2 polarization to induce metastasis of CC	CC	[79]
MeCP2	K271	P300/HDAC3	Exercise-induced Mecp2 lactylation alleviates atherosclerosis	Atherosclerosis	[121, 124]
Histone H3	K18	P300/HADC3	Promotes lipid peroxidation-induced EndMT and increases lipid burden with P300/ASF1A molecular complex	Atherosclerosis	[122]
Histone H4	K12	HADC3	Promotes atherosclerosis by TRAP1-induced metabolic reprogramming	Atherosclerosis	[125]
PCSK9	Pan-Kla	/	Promotes progression and metastasis of colon cancer	Colon cancer	[94]
AMPKα	/	/	Exacerbates NPC senescence	IVDD	[134]
HADHB	K273	/	Regulates FA metabolism in mice liver	Exercise	[66]
ACSL1	K676	/	Promotes FA metabolism and fat loss	Exercise	[66]

Mitochondrial FA synthesis (mtFAS) is a type II FAS pathway occurring in the mitochondrial matrix. The major difference between mtFAS and cytosolic FASN is that mtFAS utilizes individual enzymes for each step, whereas the latter uses different domains on a single polypeptide [40, 41]. Mitochondrial ketoacyl synthase 3-oxoacyl-ACP synthase (OXSM) catalyzes the condensation reaction, generating ketoacyl-intermediates for subsequent reduction and dehydration reactions 40]. The phosphoenolpyruvate carboxykinase 1/2 (PCK1/2) is more intricate than just gluconeogenesis, as its protein kinase activity can activate sterol regulatory element-binding proteins (SREBPs), promoting lipogenesis [42, 43]. Recently, lactate-primed KAT8 was shown to directly lactylate PCK2, enhancing its kinase activity and ultimately exacerbating hepatic ferroptosis and ischemia/reperfusion injury (IRI) by modulating OXSM-mediated mtFAS [44]. Specifically, PCK2 lactylated at K100 competitively binds to OXSM, disrupting its interaction with the ubiquitin E3 ligase Parkin. This prevents Parkin-dependent degradation of OXSM protein, thereby potentiating the production of mtFAS products, which in turn maintains OXPHOS and TCA cycle [44]. Whether the pro-glycolytic and the pro-lipogenic effects of PCKs are parallel or sequential remains to be further elucidated.

Growing evidence demonstrates connections between histone lactylation and dysregulated lipid metabolism [45, 46]. Histone H3 lysine 18 lactylation (H3K18la) upregulates expression of the m^6^A reader YTHDC1, thereby enhancing the stability of long noncoding RNA NEAT1. This further activates stearoyl-CoA desaturase (SCD) by histone acetylation, thus facilitating cancer progression [45]. SCD is a key enzyme that converts the saturated FA derived from DNL or diet into monounsaturated FA [47]. Intriguingly, H3K18la/YTHDC1/NEAT1-mediated open chromatin region of the *SCD* promoter contains the binding consensus sequence of SREBP1, suggesting chromatin alterations at the SCD locus may facilitate SREBP1 expression and subsequently activate SCD [45]. Moreover, H3K18la enrichment on the proximal promoter of *methyltransferase 3 (METTL3)* promotes SCD1 m^6^A levels, resulting in enhanced mRNA stability [48]. H3K18la also enhances total cholesterol (TC) and TG levels as well as those of lipogenesis-related proteins (FAS, acetyl-CoA carboxylase [ACC], and SREBP1) [48]. In addition, H4K12la, which is mediated by family with sequence similarity 172 member A (Fam172a), has been identified as a regulator of lipid and glucose metabolism in hypothalamic proopiomelanocortin (POMC) neurons [49]. Fam172a-specific deletion increases H4K12la levels, which are enriched in the promoter region of *peptidylglycine α-amidating monooxygenase (PAM)* to promote the synthesis of α-melanocyte stimulating hormone (α-MSH), thereby inhibiting food intake [49]. Taken together, these findings reveal that histone lactylation promotes lipid metabolism abnormalities. Furthermore, its function is not isolated but rather coordinates or competes with other PTMs to regulate lipid metabolism network.

Additionally, emerging evidence indicates a strong association between lactylation and several lipid metabolism proteins, including ATP citrate lyase (ACLY), acyl-CoA synthetase family member 2 (ACSF2), acyl-CoA synthetase short-chain family member 2 (ACSS2), FA-binding protein 5 (FABP5), lipocalin 2 (LCN2), and sterol carrier protein 2 (SCP2) [36, 50–54]. However, direct evidence demonstrating that these lactylation-driven proteins exert lipid reprogramming functions remains limited. Further research is needed to elucidate the underlying mechanisms of their effects.

### β-oxidation (FAO)

Carnitine palmitoyl transferase 2 (CPT2), located in the inner mitochondrial membrane (IMM), serves as the rate-limiting enzyme for FAO and facilitates the transport of long-chain acyl-carnitines into the mitochondrial matrix [55, 56]. Hypoxia promotes mitochondrial lactyltransferase AARS2 accumulation, leading to the lactylation of CPT2 at K457/8 and PDHA1 at K336 in the pyruvate dehydrogenase complex through a lactate- and ATP-dependent, pyrophosphate-inhibitable covalent mechanism [37]. Lactylation inactivates both enzymes and prevents LCFA transports into mitochondria efficiently, which limits FAO and reduces acetyl-CoA and ATP production, impairing OXPHOS 37]. Targeting this feedback mechanism under intracellular hypoxia may potentially enhance muscular OXPHOS and improve exercise endurance. Moreover, AARS2-triggered CPT2 lactylation results in FFA accumulation, which further activates peroxisome proliferator-activated receptor γ (PPARγ), and potentiates follicle-stimulating hormone (FSH)-mediated initiation of follicle development [57]. Furthermore, mutated AARS2 (R199C) exhibits stronger lactyltransferase activity, leading to hyperlactylation through increased lactate binding affinity due to its proximity to the substrate-binding pocket of AARS2. Additionally, PDHA1 lactylation suppresses oxidation of glycolysis/phosphopentose pathway (PPP) metabolites through TCA cycle, redirecting intermediates toward anabolic pathways. This subsequently activates proliferation-related pathways such as mTORC1 signaling, promoting granulosa cell (GC) proliferation [57]. The synergistic enhancement of GC proliferation driven by lactylation complements FSH action, jointly driving primordial follicle development.

PPARα, a ligand-activated transcription factor belonging to the nuclear steroid hormone receptor superfamily, is activated by lipid species or peroxisome proliferators and subsequently translocates to nucleus [58, 59]. There, it initiates the transcription of CPT1A and acyl-coenzyme A oxidase 1 (ACOX1), which facilitate FAO in mitochondria and peroxisomes, respectively [60]. ACOX1 primarily mediates the catabolism of very long-chain FA (VLCFA). Recent evidence indicates that PPARα enhances glioblastoma (GBM) sensitivity to temozolomide (TMZ) and reverses acquired drug resistance through H3K18la inhibition [61]. Mechanistically, TMZ activates PPARα via p38 MAPK signaling, leading to ACOX1 upregulation. ACOX1 then inhibits H3K18la by inducing ROS-dependent PKM2 downregulation. This, in turn, enhances PPARα activation via ROS-activated ASK1/p38 MAPK pathway, forming a positive feedback loop that improves TMZ therapeutic efficacy.

Additionally, lysine (K)-specific demethylase 6B (KDM6B) enhances FAO by facilitating H3K27me3 demethylation and lactylation in the *hydroxyacyl-CoA dehydrogenase α subunit (HADHA)* promoter, which upregulates HADHA expression [62]. This may be attributed to the fact that lactylation not only prevents protein ubiquitination-induced degradation but also increases enzyme activity [63, 64]. These changes promote FAO and a resultant increase in ATP, which further accelerates mineralization [62]. In contrast, HADHA lactylation at K166/728 inhibited its activity, which disturbed mitochondrial function, ATP production, and energy metabolism on cardiomyocytes [65]. Lactylation of HADHB at K273 and acyl-CoA synthetase long-chain family member 1 (ACSL1) at K676 is involved hepatic FA metabolism in mice during moderate-intensity exercise, potentially enhancing fat loss efficiency [66]. These lactylations may serve as signals to coordinate lipid metabolism; however, the underlying mechanisms remain incompletely understood and require further investigation.

### Lipolysis

Excessive FFA disrupt cellular membranes and exert cytotoxic effects [15]. To mitigate lipotoxicity, FFA are sequestered into TG, and typically stored within lipid droplets (LDs). During energy mobilization, lipolysis occurs via a cascade of cytosolic lipase-mediated hydrolysis reactions, resulting in FFA release [67]. Emerging evidence indicates that apolipoprotein C2 (APOC2) lactylation enhances lipolysis to promote FFA release, correlating with tumor metastasis and immunotherapy resistance [32]. APOC2 is lactylated at K70 and ubiquitinated at K52, K61, K70, K96, and the lactylation stabilizes it by inhibiting its ubiquitination [32]. Under a high lactate microenvironment, lactylated-APOC2 is secreted via paracrine signaling into the tumor microenvironment (TME), where it binds to highly expressed lipoprotein lipase (LPL) to form the complex that enhances TG hydrolysis, thereby producing FFA for tumor metabolism. These findings illuminate detrimental roles of lactylation in promoting lipolysis and suggest that targeting this pathway may potentially provide therapeutic strategies for diseases associated with dysregulated lipolysis.

### Lipid peroxidation and ferroptosis

Ferroptosis is a form of regulated cell death driven by intracellular iron-dependent phospholipid peroxidation. Redox signaling, lipid metabolism, and iron homeostasis collectively modulate its mechanisms. Notably, cellular lipids and lipid metabolism play a central role in its regulation, with sensitivity determined by levels of peroxidizable polyunsaturated FA (PUFA) and associated lipid metabolic enzymes [68, 69]. ACSL4 serves as a promotor in ferroptosis execution though shaping lipid composition [70]. It catalyzes the esterification of arachidonic acid (AA) into phosphatidylethanolamines (PEs), with AA-PE serving as the primary substrate for iron-induced peroxidation in ferroptosis [71]. Recent researches indicate lactylation regulates lipid peroxidation by altering the activity and binding capacity of proteins interacting with ACSL4 [72–74]. For example, H3K18la enhances METTL3-mediated m^6^A modification of ACSL4, increasing PUFA levels and ferroptosis sensitivity [72]. Notably, lactate promotes ACSL4 lactylation at K412, boosting its enzymatic activity and facilitating ACSL4 dimer formation, which is essential for its activation [73]. ACSL4 inhibition results in lipidomic changes in lactate-treated nucleus pulposus cells (NPCs), particularly affecting long-chain PUFA-containing phospholipids [73]. Furthermore, H3K18la enrichment on the *HIF1A* promoter enhances the expression of ACSL4, lysophosphatidylcholine acyltransferase 3 (LPCAT3), and arachidonate 15-lipoxygenase (ALOX15), which are critical for the synthesis and oxidation of specific phospholipids, thereby contributing to ferroptosis [74]. Moreover, lactylation of Sun domain family member 2 (NSUN2) suppresses lipid peroxidation and enables cancers to evade ferroptosis in acidic TME [75]. Collectively, these findings indicate lactylation regulates lipid peroxidation by modifying key enzymes or regulatory proteins in this pathway.

### Lactylation of lipids

Vacuolar protein sorting protein 34 (Vps34) lactylation at K356/781 enhances its binding to Beclin1, Atg14L, and UVRAG, thereby increasing lipid kinase activity and promoting macroautophagy/autophagy and endo-lysosomal degradation [76]. Vps34 phosphorylates phosphatidylinositol (PI) to generate phosphatidylinositol-3-phophate (PI3P), which is essential for intracellular lipid signaling and membrane dynamics [77]. Similarly, rubicon autophagy regulator (RUBCN) lactylation at K33 strengthens its interaction with Vps34, increasing lipid kinase activity and maintaining PI3P levels on LAPosomes [78]. These findings delineate a lactylation-dependent mechanism for Vps34 regulation, providing a potential target for autophagy-related diseases. Glycerol-3-phosphate dehydrogenase 2 (GPD2), a key enzyme in glycerophospholipid metabolism, is upregulated by H3K18la in macrophages, although the precise molecular mechanism requires further investigation [79].

## Lactylation-mediated control of cholesterol metabolism

Cholesterol is an essential constituent of the lipid bilayer in cellular membranes, derived from both endogenous biosynthesis and exogenous uptake via lipoproteins. It maintains membrane permeability and fluidity, modulates membrane protein functions, and participates in membrane trafficking and transmembrane signaling. De novo cholesterol biosynthesis involves multi-step enzymatic reactions in the mevalonate (MVA) pathway. Thereafter, cholesterol can be converted into esters or oxysterols, serving as precursors for bile acids and steroid hormones [80–82]. Emerging evidence indicates that lactylation is closely linked to cholesterol homeostasis [83–86].

### Cholesterol synthesis

Cholesterol biosynthesis is a complex and highly regulated metabolic process. Dysregulated production of MVA pathway metabolites modulates signaling pathways in cancers and contributes to cellular transformation [87]. Hyperactivated AKT phosphorylates gluconeogenic enzyme PCK1, and converts its function into protein kinase. Consequently, phosphorylated PCK1 (pPCK1) phosphorylates LDHA, enhancing lactate production and subsequently inducing SPRING lactylation at K82 [83]. SPRING is a recently identified regulatory protein that modulates hepatic SREBP cleavage-activating protein (SCAP)-SREBP movement [88, 89]. In cholesterol-rich conditions, the ER cholesterol sensor SCAP interacts with insulin-induced gene protein (Insig), retaining the SCAP-SREBP complex within ER and inhibiting cholesterol synthesis. In a cholesterol-limited environment, SCAP-SREBP are dissociated from Insig, sorted into the COPII vesicles and transported to Golgi. There, SREBP undergoes proteolytic cleavage, releasing its mature form, which translocates to nucleus to initiate cholesterol biosynthesis [90]. SPRING lactylation mediated by pPCK1-pLDHA axis enhances cholesterol metabolism by promoting the retrograde transport of SCAP from Golgi back to ER, thus improving trafficking efficiency of the SCAP-SREBP complex [83]. This mechanism results in elevated levels of MVA and its derivates (CoQ_10_H_2_, MK4), revealing a distinct mechanism of lipogenesis. Conversely, inhibition of SPRING lactylation impairs SREBP1/2 maturation, reduces SREBP2-driven MVA flux [83]. Collectively, KAT7-mediated SPRING lactylation positively regulates cholesterol metabolism by modulating SCAP trafficking and serves as a key intermediary in the metabolic reprogramming of glycolysis and the MVA pathway driven by pPCK1-pLDHA axis.

Acetyl-CoA acyltransferase 2 (ACAT2), also known as acetoacetyl-CoA thiolase, plays a crucial role in cholesterol biosynthesis by catalyzing acetoacetyl-CoA formation from acetyl-CoA and contributing to the isoprenoid biosynthesis pathway. Histone lactylation, particularly H3K18la, directly enhances ACAT2 transcription, thereby enhancing cholesterol biosynthesis. Moreover, ACAT2 stabilizes its own expression by acetylating mitochondrial carrier homolog 2 (MTCH2), which inhibits MTCH2 degradation and consequently reduces mitochondrial OXPHOS [84]. These mechanisms establish a positive feedback loop involving lactate/H3K18la/ACAT2, highlighting the critical role of lactylation in driving cholesterol metabolic reprogramming. Notably, ACAT2 has been identified as a lactylation writer that facilitates the glutamate-cysteine ligase (GCL) modifier (GCLM) lactylation at K34, enhancing GCL enzymatic activity and GSH synthesis, thereby resulting in ferroptosis resistance in KRAS^G12D^-mutant cancers [86].

### Cholesterol metabolic conversion

Progesterone synthesis represents one of the important pathways in cholesterol biotransformation. Steroidogenic acute regulatory protein (STAR) facilitates the transport of free cholesterol from cytoplasm to IMM, providing the substrate for cytochrome P450 family 11 subfamily A member 1 (CYP11A1) [91]. CYP11A1 then catalyzes the side-chain cleavage of cholesterol to produce pregnenolone, which is subsequently transported to ER and converted into progesterone [92]. These coordinated reactions ensure efficient synthesis and secretion of progesterone in physiological conditions. Recently, lactylation was shown to facilitate the transformation of cholesterol to progesterone and to modulate the expression of associated genes under hypoxia [85]. Specifically, hypoxia enhances hCG-induced H3K18la, activating the transcription of STAR and CYP11A1 and stimulating progesterone production during GC luteinization. Additionally, cAMP response element-binding protein (CREB) lactylation at K136 is also required for hCG-induced GC luteinization [85]. These suggests that both histone and non-histone lactylation synergistically modulate metabolic reprogramming during the initial phase of cholesterol transformation into steroid hormones, shedding new light on the significance of metabolic–epigenetic crosstalk in reproductive biology.

### Cholesterol transport

Cholesterol transport is a complex and dynamic process, primarily involving absorption, synthesis-mediated distribution, and reverse transport for recycling. Recent studies have demonstrated the H3K18la/ACAT2/small extracellular vesicles (sEVs)-cholesterol axis plays a critical role in TME reprogramming [84]. H3K18la-driven ACAT2 expression promotes cholesterol transport via sEVs, which further influences the polarization of tumor-associated macrophages (TAMs) toward the M2-like phenotype, suppresses CD8^+^ T-cell activity, and contributes to establishing the immunosuppressive TME [84]. This immunosuppressive effect is closely associated with cholesterol accumulation. Studies also have indicated that apolipoproteins, including APOA1, APOC1, and APOC3, exhibit increased lactylation levels, which play important roles in cholesterol transport [93]. However, their protein levels are reduced due to lactylation-induced activation of proteasomal degradation rather than direct regulation of transcription or translation. This “high lactylation–low function” phenomenon establishes a pathological mechanism, suggesting lactylation might provide novel insights into these metabolic disturbances. Additionally, proprotein convertase subtilisin/kexin type 9 (PCSK9) regulates lipoprotein homeostasis and facilitates cancer metastasis partially through lactylation [94]. H4K12la regulates the expression of ATP binding cassette subfamily C members (ABCC2, ABCC3, and ABCC10). Their enhanced activity increases the efflux of drugs and metabolic products, contributing to chemotherapy resistance [95]. Although the underlying mechanisms require further elucidation, these findings collectively suggest lactylation regulates cholesterol homeostasis in relevant diseases and may facilitate the identification of potential targets within the cholesterol metabolic profiles.

## Lactylation in lipid-related diseases

**Fig. 3:**
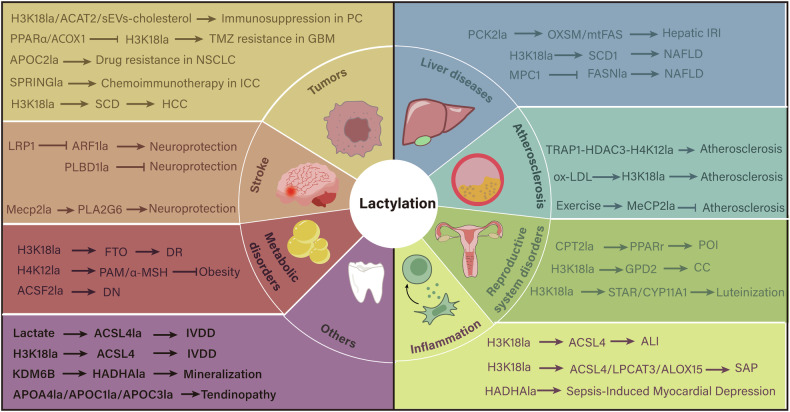
Known pathways by which lactylation regulates lipid metabolism in disease. Lactylation is closely associated with the occurrence and development of various lipid metabolism-related diseases and could potentially represent a therapeutic target.

Lactylation functions as a “double-edged sword” and is involved in the development of lipid-related diseases including cancers, metabolic diseases, and cardio-cerebrovascular diseases [17, 18]. However, the related mechanisms remain unclear. Recently, several innovative methods, including genetic code expansion-based and probe-targeted workflows, have been applied to characterize lactylation [96–99]. These advancements have substantially enhanced our understanding of the challenges and advances of lactylation in lipid-related diseases (Fig. 3).

### Liver diseases

Non-alcoholic fatty liver disease (NAFLD) is characterized by hepatic accumulation of fat, with abnormal DNL activation serving as a critical mechanism in its pathogenesis [100, 101]. Both histone and non-histone lactylation contribute to its progression by regulating lipid reprogramming [102]. FASN lactylation is regarded as the main mechanism through which mitochondrial pyruvate carrier 1 regulates lipid synthesis in NAFLD hepatocytes [34]. In contrast, histone lactylation, particularly H3K18la, increases TC and TG levels as well as key indicators of FA synthesis, and collaborates with m^6^A modification to promote NAFLD progression [48]. High alcohol-producing *Klebsiella pneumoniae* induce histone lactylation, leading to lipid accumulation and mitochondrial dysfunction in NAFLD [103]. Collectively, FASN lactylation inhibits DNL to alleviate NAFLD progression, whereas H3K18la aberrantly regulates lipid metabolism reprogramming to exacerbate NAFLD. These results provide a foundation for investigating the paradox of lactylation in lipid metabolism with the goal of identifying potential biomarkers for monitoring NAFLD and related diseases.

Hepatic IRI is a significant risk factor for primary graft dysfunction or non-function and acute/chronic rejection following liver transplantation (LT) surgery [104]. Hepatocyte ferroptosis dominates early injury, whereas macrophage pyroptosis and necroptosis predominate in steatotic livers during late phases [105]. Recently, lactylation has emerged as a promising therapeutic target for managing IRI, and direct links among hyperlactatemia, ferroptosis, and hepatic IRI have been established [44, 106]. PCK2 lactylation exacerbate hepatic ferroptosis and IRI via mtFAS reprogramming, making mtFAS pathway a promising therapeutic target. However, no inhibitors of mtFAS pathway are available for clinical application. Currently, PCK2-targeted agents hold promise as a therapeutic strategy to inhibit hepatic ferroptosis and IRI in patients with hyperlactatemia during LT. The implementation of these novel strategies may not only enhance clinical outcomes but also facilitate the utilization of marginal liver grafts, thereby expanding donor pool for life-saving LT.

### Tumors

Overactive lipid metabolism is a typical feature of tumors and a key driver of tumor progression [32]. Lipid reprogramming influences tumor initiation, development, invasion, metastasis, and even therapy resistance by modulating oncogenic signaling pathways [107, 108]. Elevated lactate levels are a prominent feature in TME, and currently, lactylation is recognized as a widespread modification affecting almost all abnormally regulated metabolic pathways in cancers [35, 45, 46, 109]. Accordingly, the intricate mechanisms between lactylation and lipid metabolism have garnered significant attention. In particular, the effects of lactylation in cancers predominantly appear to promote tumorigenesis and development [110]. For example, elevated lactylation, including histone and non-histone lactylation, have been shown to promote the proliferation and metastasis of hepatocellular carcinoma (HCC), pancreatic cancer (PC), and pancreatic ductal adenocarcinoma (PDAC), which are associated with poor survival [31, 46, 86, 111]. Lactylation may serve as a diagnostic marker for tumors [112–114]. Whether lactylation exhibits inhibitory effects on tumor growth, migration, or invasion remains to be elucidated.

Although chemoimmunotherapy regimens have shown modest success, persistent resistance and low response rates underscore the urgent needs for novel therapeutic strategies for specific cancers. Lactylation plays a critical role in anti-tumor drug resistance, acting both as an inducer and a target for reversing resistance [115–117]. In non-small cell lung cancer (NSCLC), anti-APOC2 K70 lactylation antibodies neutralize extracellular APOC2, suppressing tumor growth and potentially offering combinational approaches for patients with NSCLC and other APOC2-associated diseases [32]. In intrahepatic cholangiocarcinoma (ICC) patients, targeting pPCK1-pLDHA-SPRINGla axis with simvastatin effectively inhibits the MVA pathway, resulting in enhanced chemoimmunotherapy efficacy [83]. Moreover, targeting H3K18la/ACAT2 enhances the anti-PD-1 therapy response in PC, providing novel therapeutic strategies by linking lactylation, cholesterol metabolism reprogramming, and immune modulation [84]. The KRAS^G12D^-driven metabolic adaptation linking GCLM lactylation to ferroptosis resistance highlights the potential of ACAT2 inhibition as a therapeutic strategy [86]. Interestingly, compared with other lactylations, H3K18la may play a more important role in mediating cancer progression, metastasis, and drug resistance because of its enrichment in the promoters of a subset of active genes [63, 118, 119]. For example, H3K18la regulates ferroptosis and contributes to doxorubicin resistance in triple-negative breast cancer [120]. Moreover, PPARα contributes to TMZ-induced GBM growth arrest by inhibiting H3K18la, and targeting PPARα with lipid-lowering agent gemfibrozil can effectively reverse the acquired resistance via H3K18la suppression [61].

Thus, clinical researchers have increasingly focused on the interplay between lactylation and lipid metabolism in cancer. This focus aims to address key questions to provide new insights into tumor progression and drug resistance mechanisms as well as to identify potential drug targets and advance personalized treatment strategies.

### Atherosclerosis

Atherosclerosis occurs as a result of multiple risk factors, including lipid dysregulation, endothelial dysfunction, foam cell formation, chronic inflammation, vascular smooth muscle cell (VSMC) proliferation, apoptosis, and necrosis. Abnormal lipid metabolism triggers endothelial dysfunction, which drives macrophage and VSMC-derived foam cell formation, ultimately leading to atherosclerotic plaque development [102]. Increasing evidence suggests that lactylation influences lipid metabolic imbalance in atherosclerosis by modulating transcription factors, metabolic regulatory proteins, and inflammatory response [121–123]. For instance, exercise-induced methyl-CpG-binding protein 2 (MeCP2) lactylation at K271 in endothelial cells inhibits atherosclerotic lesions and lipid deposition in Apoe^KO^ mice [121, 124]. H3K18la, together with P300 and anti-silencing function 1 A (ASF1A), promotes ox-LDL-induced endothelial-to-mesenchymal transition (EndMT) in atherosclerosis; however, Asf1a^ECKO^-induced H3K18la inhibition alleviates the lipid burden in total artery and root sections of Apoe^KO^ mice [122]. Conversely, H3K18la initiates local repair in MCT4-deficient macrophages to alleviate atherosclerosis [123]. Moreover, tumor necrosis factor receptor-associated protein 1 (TRAP1) deficiency-induced H4K12la inhibition reduces lipid accumulation, shrinking the plaque area and necrotic core in the aortic root and indicating a mitonuclear communication mechanism in atherosclerosis [125]. Additionally, Huazhuo Tiaozhi granule achieves hypolipidemic properties partially via H2BK6la and H4K80la [126]. Overall, these findings reveal the multifaceted mechanisms by which lactylation can either accelerate lipid deposition and disease progression, or alleviate atherosclerosis depending on different context. Investigating the dual role of lactylation may therefore hold significant promise for developing innovative anti-atherosclerosis strategies.

### Metabolic disorders

In metabolic disorders, the imbalance between uptake or synthesis and consumption of FA results in accumulation of lipid intermediates, leading to cellular dysfunction in metabolically active tissues including kidney, brain, and skeletal muscle [100]. Lactylation is closely associated with metabolic diseases [34, 49]. Fam172a-mediated H4K12la in POMC neurons reduces adiposity and body weight, improving glucose tolerance and insulin sensitivity in mice. Conversely, H4K12la inhibition causes an obesity-like phenotype [49]. Hypothalamic histone lactylation in central melanocortin system may provide new therapeutic directions for diet-induced obesity. Systemic and tissue-specific alterations in lipid metabolism are recognized as key factors in the pathology of diabetes and its complications, such as diabetic nephropathy (DN) and retinopathy (DR) [127]. H3K18la upregulates fat mass and obesity-associated protein (FTO), aggravating microvascular anomalies in DR [128]. ACSF2 lactylation at K182 mediates mitochondrial dysfunction in DN [36]. Furthermore, lactylation correlates with insulin resistance in human skeletal muscle [129]. Collectively, increasing research is focusing on the relationship between lactylation and lipid metabolism in metabolic disorders, seeking ways to improve patient outcomes through targeting of lactylation.

### Degenerative musculoskeletal diseases

Accumulating evidence indicates degenerative musculoskeletal diseases accompanied by a spectrum of lipometabolic disturbances [130–132]. In rotator cuff tendinopathy, lactylation of key apoliproteins in tendons could potentially serve as diagnostic markers or therapeutic targets for restoring metabolic homeostasis and mitigating tendon degeneration [93]. In intervertebral disc degeneration (IVDD), NPCs exhibit abnormal glycolytic activity, inducing lactate accumulation [133]. Lactylation increases the susceptibility of NPCs to ferroptosis by enhancing ACSL4–phospholipid peroxidation axis, offering an alternative strategy for IVDD treatment [73]. AMPKα lactylation suppresses its phosphorylation to exacerbate NPC senescence in IVDD [134]. Moreover, LDHB promotes ACSL4 expression via histone lactylation, contributing to osteoarthritis (OA) progression [135]. Lactylation serves as a bridge linking lipid metabolism reprogramming to musculoskeletal degeneration and provides a novel target for improving outcomes of these diseases.

### Inflammatory diseases

Lactylation exerts either pro-inflammatory or anti-inflammatory effects depending on the metabolic profile and cell type. Furthermore, KL-la and KD-la display distinct effects: KL-la promotes M2 macrophage polarization, whereas KD-la correlates with increased inflammatory cytokine production [1, 136]. In the context of lactylation and lipid metabolism, lactylation contributes to inflammation in acute lung injury (ALI) and severe acute pancreatitis (SAP). ALI associated with sepsis is a severe complication in sepsis patients with high mortality. During ALI, inflammation and oxidative stress lead to energy depletion, which impairs FAO, increases expression of proteins involved in FA uptake and transport, enhances FA synthesis and LD accumulation [137]. Reversing the expression of key enzymes in FA metabolism can effectively mitigate ALI severity. In ALI/ARDS patients, local lactate levels positively correlate with the severity of lung injury. The H3K18la/METTL3/ACSL4 axis contributes to mitochondrial damage and ferroptosis in ALI via lipid peroxidation [72]. Furthermore, HADHA lactylation promotes sepsis-induced cardiac dysfunction, highlighting its potential as a novel therapeutic strategy for sepsis-associated myocardial depression [65]. Lipid metabolism also significantly impacts the development and progression of SAP, a life-threatening condition with high mortality, thereby closely influencing both its occurrence and severity [138]. H3K18la induces HIF1A transcriptional activation and exacerbates SAP through ACSL4/LPCAT3/ALOX15 pathway [74]. Targeting lactylation or key enzymes may mitigate disease severity and offer new strategies for early detections.

### Reproductive system disorders

Primary ovarian insufficiency (POI) severely impacts women’s fertility, with dysregulated glucose and lipid metabolism serving as a causal factor [57]. Follicular development is initiated by GC proliferation, which is activated by FSH and sustained through anabolic metabolism. Lactylation can initiate follicle development by promoting GC proliferation [57, 85]. Under hypoxia, hCG stimulation upregulates H3K18la and CREB lactylation in GCs to promote luteinization [85]. Moreover, CPT2 lactylation leads to FFA accumulation and PPARγ activation, which potentiates FSH-induced primordial follicle development [57]. However, excessive lactylation accelerates folliculogenesis, ultimately inducing POI. Inhibiting lactylation prevents POI traits, making it a promising intervening target for POI. Furthermore, β-alanine shows potential as a therapeutic agent for POI prevention and treatment, potentially by reducing lactylation. Preeclampsia (PE) is a dangerous complication during pregnancy, and lipid metabolism abnormalities represent significant risk factors [139]. Lactylation is closely associated with lipid-related proteins in PE, including HMG-CoA synthase 2 (HMGCS2), scavenger receptor class B member 1 (SCARB1), APOC3, APOC2, LDL receptor-related protein 2 (LRP2) [140]. These findings enhance the epigenetic understanding of reproductive disorders and their lactylation profiles in lipid metabolism. Further studies are required to elucidate the underlying mechanisms.

### Ischemic stroke

Acute ischemic stroke often results in devastating and permanent neurological damage and is a leading cause of morbidity and mortality worldwide. Lipids and lipid intermediates play significant roles in maintaining normal brain structure and function. However, lipid metabolism dysfunctions are closely associated with ischemic stroke. Alterations in neuronal phosphatidylcholine remodeling and FFA–LD coupling between neurons and astrocytes are implicated in its pathogenesis [141, 142]. LRP1, a multifunctional transmembrane receptor in the LDLR family, suppresses ADP-ribosylation factor 1 (ARF1) K73 lactylation in astrocytes, thereby mitigating cerebral ischemic injury via mitochondria-mediated astrocyte–neuron crosstalk [143]. The LRP1/ARF1 lactylation axis is a potential therapeutic target for ischemic stroke and other neurodegenerative disorders, but further research is needed to elucidate the mechanisms of lipid metabolism coordination between neurons and astrocytes [143]. Moreover, phospholipase B domain-containing protein 1 (PLBD1) lactylation at K155 exacerbates ischemic stroke, whereas MeCP2 lactylation at K210/249 alleviates it [144, 145]. MeCP2 lactylation represses apoptosis-associated genes, including *Pdcd4* and *Pla*_*2*_*g6*, thereby reducing neuronal apoptosis. Phospholipase PLA_2_G6 selectively hydrolyzes glycerophospholipids to release FFA [146]. However, the neuroprotective effects of MeCP2 lactylation on PLA_2_G6 via lipid metabolism require further investigation.

### Others

The KDM6B/HADHA lactylation/FAO pathway has been identified as a critical mechanism in mineralization, representing a new therapeutic target for cementum regeneration [62]. Additionally, protein disulfide-isomerase (P4HB) lactylation also has been identified as a new target of radiation-induced heart disease [147].

## Conclusions

Lactylation functions as a multifaceted regulator in major lipid metabolism pathways, thereby influencing the progression of various diseases. Systematically investigating the lipid metabolizing enzymes, targets, and associated signal transduction pathways will deepen comprehension of their intricate interactions. This, in turn, will facilitate the development of more accurate biomarkers for disease progression and personalized therapies. However, several challenges remain to be addressed. First, lipid metabolism does not operate in isolation but rather interacts with other metabolic pathways, forming a dynamic and complex network. Different diseases exhibit distinct lipid signatures across multiple cellular types, and their interactions significantly impact lipid metabolism and lactylation. Second, lactylation interacts with other PTMs in lipid metabolism reprogramming (Table 3). Their potential synergistic or competitive relationships, and the functional consequence of their differing ratios, may influence disease progression and prognosis. Additionally, whether different forms of lactylation interact during disease progression remains unclear. Third, none of the currently identified enzymes exhibit strict specificity for lactylation [148, 149], and their essential enzymatic parameters remain unknown, raising concerns about identifying the exact conditions that effectively catalyze the process. In conclusion, the adaptability of lactylation to metabolic shifts and its influence on metabolic reprogramming render lactylation a promising therapeutic target.

**Table 3:** Lactylation events that act in combination with other PTMs to regulate lipid metabolism.

Lactylation	Other PTMs	Functions	Diseases	Refs
PCK2 K100	Ubiquitination	Prevent Parkin-mediated polyubiquitination of OXSM	LT	[44]
H3K18la	m^6^A modification and acetylation	Promote m^6^A-modified NEAT1 stability, activate SCD by histone acetylation	HCC	[45]
H3K18la	m^6^A modification	Promote METTL3-mediated SCD1 m^6^A levels	NAFLD	[48]
H3K18la	m^6^A modification	Promote METTL3-mediated m^6^A modification on ACSL4	ALI	[72]
HADHA K519	Methylation	KDM6B-mediated HADHA demethylation/ lactylation regulates cementogenesis	Periodontitis	[62]
NSUN2 K508	m^5^C modification	Promote GCLC m^5^C formation	GC	[75]
Mecp2 K271la	Methylation	Exercise ameliorates atherosclerosis via Mecp2 k271la-H3k36me3/RUNX1	Atherosclerosis	[124]
AMPKα lactylation	Phosphorylation	AMPKα lactylation inhibits its phosphorylation	IVDD	[134]
H3K18la	ACSL4 lactylation	H3K18la promotes ACSL4 transcription, while lactate directly stimulates non-histone lactylation of ACSL4 at K412	IVDD	[73]
H3K18la	MTCH2 acetylation	H3K18la-induced ACAT2 transcription promotes MTCH2 K100 acetylation, which disrupts OXPHOS and establishes a positive feedback loop	PC	[84]
SPRING lactylation	Phosphorylation	pPCK1 binds to and phosphorylates LDHA, further promoting SPRING lactylation at K82 and thereby enhancing MVA pathway activity	ICC	[83]

## Data Availability

We confirm that the text, tables and figures in this review are original. There is no original research data disclosed or included in this review.
